# Supplementary material for: Causal Relationship between Aging and Anorexia Nervosa: A White-Matter-Microstructure-Mediated Mendelian Randomization Analysis
Source: Biomedicines. 2024 Aug 16;12(8):1874. doi: 10.3390/biomedicines12081874 (PMC11351342; doi:10.3390/biomedicines12081874)
Supplement: Supplementary file 1 [file biomedicines-12-01874-s001.zip › Supplementary Figure/Figure S4/Singe SNP plot of MR analysis for TL to ubm-b-1550.pdf]

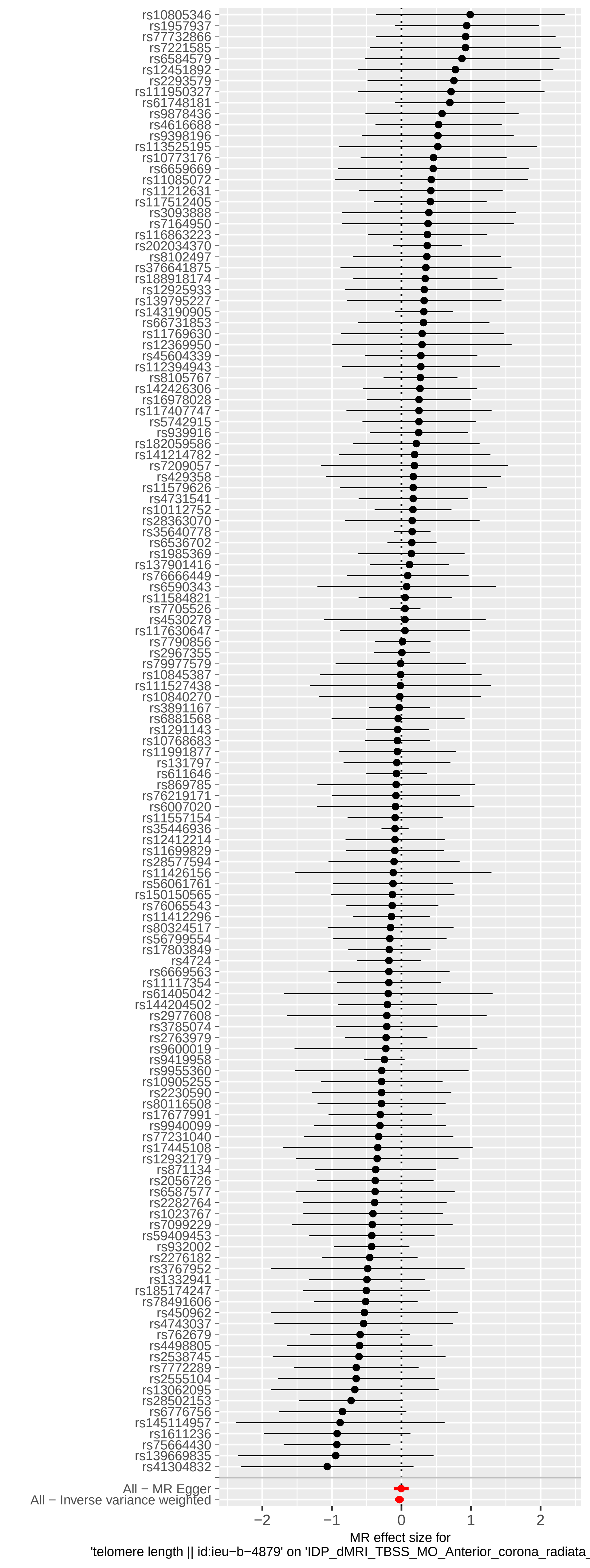
rs10805346
rs1957937
rs77732866
rs7221585
rs6584579
rs12451892
rs2293579
rs111950327
rs61748181
rs9878436
rs4616688
rs9398196
rs113525195
rs10773176
rs6659669
rs11085072
rs11212631
rs117512405
rs3093888
rs7164950
rs116863223
rs202034370
rs8102497
rs376641875
rs188918174
rs12925933
rs139795227
rs143190905
rs66731853
rs11769630
rs12369950
rs45604339
rs112394943
rs8105767
rs142426306
rs16978028
rs117407747
rs5742915
rs939916
rs182059586
rs141214782
rs7209057
rs429358
rs11579626
rs4731541
rs10112752
rs28363070
rs35640778
rs6536702
rs1985369
rs137901416
rs76666449
rs6590343
rs11584821
rs7705526
rs4530278
rs117630647
rs7790856
rs2967355
rs79977579
rs10845387
rs111527438
rs10840270
rs3891167
rs6881568
rs1291143
rs10768683
rs11991877
rs131797
rs611646
rs869785
rs76219171
rs6007020
rs11557154
rs35446936
rs12412214
rs11699829
rs28577594
rs11426156
rs56061761
rs150150565
rs76065543
rs11412296
rs80324517
rs56799554
rs17803849
rs4724
rs6669563
rs11117354
rs61405042
rs144204502
rs2977608
rs3785074
rs2763979
rs9600019
rs9419958
rs9955360
rs10905255
rs2230590
rs80116508
rs17677991
rs9940099
rs77231040
rs17445108
rs12932179
rs871134
rs2056726
rs6587577
rs2282764
rs1023767
rs7099229
rs59409453
rs932002
rs2276182
rs3767952
rs1332941
rs185174247
rs78491606
rs450962
rs4743037
rs762679
rs4498805
rs2538745
rs7772289
rs2555104
rs13062095
rs28502153
rs6776756
rs145114957
rs1611236
rs75664430
rs139669835
rs41304832
All − MR Egger
All − Inverse variance weighted
−2
−1
0
1
2
MR effect size for
'telomere length || id:ieu-b-4879' on 'IDP_dMRI_TBSS_MO_Anterior_corona_radiata_
